# Ge-Gen-Jiao-Tai-Wan Affects Type 2 Diabetic Rats by Regulating Gut Microbiota and Primary Bile Acids

**DOI:** 10.1155/2021/5585952

**Published:** 2021-04-16

**Authors:** Han Chen, Ye Yao, Wenbo Wang, Dongsheng Wang

**Affiliations:** ^1^Institute of Integrated Traditional Chinese and Western Medicine, Xiangya Hospital, Central South University, Changsha 410008, China; ^2^Hunan Key Laboratory of Traditional Chinese Medicine for Gan of State Administration, Central South University, Changsha 410008, China

## Abstract

The Ge-Gen-Jiao-Tai-Wan (GGJTW) formula has been used to treat type 2 diabetes mellitus (T2DM) in China for a long time. Our previous study has proved that GGJTW could alleviate the type 2 diabetic symptoms, but the underlying mechanisms are still unclear. This study aimed to investigate the changes in gut microbiota and primary bile acids (PBAs) to determine the potential mechanisms of GGJTW in treating T2DM.The fecal transplant method and pseudogerm-free rats were used in our study.The16S rRNA gene sequencing method was used to analyze the changes in the intestinal flora, and PBAs in the colon contents were detected. Finally, the expression of farnesoid *X* receptor (FXR), *G* protein-coupled membrane receptor 5 (TGR5), and glucagon-like peptide-1 (GLP-1) was assessed. Following GGJTW treatment, we observed a decrease in blood glucose levels and improvements in glucose tolerance and serum lipid levels. Furthermore, we found that GGJTW could regulate the composition of the gut microbiota and upregulate the diabetic beneficial phylum *Firmicutes* and bile-acid-related genus *Lactobacillus*. PBAs in the colon contents were increased in the GGJTW-treated group, accompanied by upregulated expression of the bile acid receptors FXR and TGR5 and increased concentrations of GLP-1. These results indicated that GGJTW could alleviate symptoms of type 2 diabetic rats by regulating the gut microbiota, promoting the production of PBAs, and upregulating the PBA-FXR/TGR5-GLP-1 pathway.

## 1. Introduction

Type 2 diabetes mellitus (T2DM) is a metabolic disease that is characterized by hyperglycemia and results from insulin resistance and insufficient insulin secretion [1].The incidence of T2DM is rapidly increasing due to changes in lifestyle and habits, including increased calorie intake and decreased physical exercise, accompanied by increasing burdens on medical services [2, 3]. Currently, the main treatment for T2DM is oral medications and insulin supplements. However, the side effects, such as liver problems and lactic acidosis, are noted [4]. Therefore, investigating new therapeutic strategies for T2DM has become an attractive research hotspot.

Traditional Chinese medicines (TCMs) have been used in China to treat diseases for thousands of years and are characterized by the holistic concept of TCM theory. Most of the Chinese medicines were formulated by “Yin Yang theory,” “Wu Xing theory,” and other TCM theories, and some Chinese medicinal formulas were created by the experience of the ancient doctors. Recently, large numbers of experimental studies have revealed that TCMs are effective in treating numerous kinds of diseases. For examples, Xu-Fu-Zhu-Yu decoction is useful for treating cardiovascular disease [5, 6], Yin-Chen-Wu-Ling power has the ability to diminish hyperlipidemia [7, 8], and Da-Huang-Ze-Xie and Da-Chai-Hu decoctions are beneficial for nonalcoholic fatty liver disease [9, 10]. Moreover, the therapeutic effects of TCMs on T2DM have gained growing attention worldwide. Many Chinese herbs, including ginseng, *Rhizomacoptidis,* and bitter melon, have the ability to improve the insulin resistance, decrease the apoptosis of pancreatic *β* cell, and increase the secretion of insulin [11]. Chinese herbal formulas, such as Shen-Zhu-Tiao-Pi granules [12], Sheng-Pu-Huang-Tang [13] and San-Huang-Tang [14], are also promising for treating T2DM.

Recently, a number of studies have suggested a link between gut microbiota and T2DM, indicating that the gut microbiota plays a vital role in T2DM and might become a potential drug target [15–19]. Because Chinese herbs have multiple components, it is difficult to study the mechanisms of TCMs in treating diseases. However, many components in Chinese herbs, such as polysaccharides [2, 18], flavonoids [20], and alkaloids [19, 21], have been documented to modulate the composition of the gut microbiota. Therefore, mechanisms of regulating the gut microbiota might exist for TCMs to treat T2DM. For example, Ge-Gen-Qin-Lian decoction was found to have the ability to enrich the amounts of beneficial bacteria in fecal samples of T2DM patients [22], and T2DM rats treated with Xie-Xin-Tang regulate the composition of gut microbiota, especially short-chain fatty acid production and anti-inflammatory bacteria [23].

Primary bile acids (PBAs), including cholic acid (CA), taurocholic acid (TCA), glycocholic acid (GCA), chenodeoxycholic acid (CDCA), taurochenodesoxycholic acid (TCDCA), and glychenodeoxycholic acid (GCDCA), are synthesized in the liver and are regulated by gut microbiota [24, 25]. Bile acids regulate and activate numerous metabolic pathways in the host via farnesoid *X* receptor (FXR) and *G*-protein-coupled membrane receptor 5 (TGR5), which increases the expression of glucagon-like peptide-1 (GLP-1) [24, 25]. The GLP-1 analogues, such as liraglutide, albiglutide, and exenatide, are widely used in the treatment of T2DM [26].

Ge-Gen-Jiao-Tai-Wan (GGJTW) is comprised of Kudzu root (Ge-Gen in Chinese), *Rhizoma coptidis* (Huang-Lian in Chinese), and cinnamon (Rou-Gui in Chinese), and it has been a classic formula for the treatment of T2DM in Xiangya Hospital for many years. Kudzu root, *Rhizoma coptidis,* and cinnamon are famous Chinese herbs and are mentioned in the Pharmacopoeia of the People's Republic of China, and all of them were first recorded in an old TCM book “Shen Nong Ben Cao Jing” in the Han Dynasty. GGJTW is derived from a well-known TCM formula, which is named “Jiao-Tai-Wan.” “Jiao-Tai-Wan” was first mentioned in a classic medical book “Han-Shi-Yi-Tong” in the Ming dynasty and comprised of Kudzu root and *Rhizoma coptidis*. We found that “Jiao-Tai-Wan” plus cinnamon had a better therapeutic effect to treatment of T2DM. Our previous animal study has proven that GGJTW could significantly alleviate T2DM symptoms, and UHPLC-QTOF/MS-based untargeted metabolomics analysis found that the most critical pathway was the PBA biosynthesis pathway involved in upregulation of bile acids [27]. Furthermore, our another study found that berberine, which is abundant in *Rhizoma coptidis* (a main component of GGJTW), could reduce the blood glucose and modulate the gut microbiota [18]. To further investigate the potential mechanisms of GGJTW in treatingT2DM, we used high-throughput 16S rRNA gene sequencing technology to identify the intestinal flora, and the Spearman correlation coefficient was utilized to analyze the relationship between the gut flora and T2DM-related biomarkers. Finally, the expression of the PBA-related pathway was detected to explore the possible mechanism of GGJTW in improving T2DM.

## 2. Methods and Materials

### 2.1. Chemicals and Reagents

Streptozotocin (STZ, batch number: 415G048, purity >98%; source: St. Louis, MO, USA) was purchased from Sigma-Aldrich Co., Ltd. Metformin (batch no. S24O8G46081, purity:98%, source: Beijing, China) was obtained from Solarbio Science and Technology Co., Ltd. Citric acid and sodium citrate (analytical reagent, batch nos. 20180512 and 20180607, source: Shanghai, China) were purchased from Sinopharm Chemical Reagent Co., Ltd. Vancomycin, neomycin, and metronidazole (analytical reagent, batch nos. 20180501, 20180402, and 20180511, source: Shanghai, China) were also purchased from Sinopharm Chemical Reagent Co., Ltd. STZ was dissolved in the freshly prepared citric acid and sodium citrate buffer (0.1 M, pH 4.5). A high-fat diet was obtained from Beijing Keao Xieli Feed Co., Ltd. (Beijing, China), and the nutritional ingredients were prepared as described in our previous study, resulting in 66.5% regular fodder, 20% sucrose, 10% lard, 2.5% cholesterol, and 1% bile salt [27]. The GGJTW formula includes Kudzu root (*Pueraria lobata (Willd.) Ohwi* root, batch no. 2018091001, source: Hunan, China), *Rhizoma coptidis* (*Coptis chinensis Franch*, batch no. 2018092516, source: Sichuan, China), and cinnamon (*Cinnamomum cassia (L.) J. Presl.*, batch no. 2018100318, source: Guangxi, China) in a ratio of 6 : 2 : 1. All herbs were purchased from the Dispensary of Traditional Chinese Medicine, Xiangya Hospital, Central South University (Hunan, China). The extracts of GGJTW were obtained from our laboratory and prepared as previously described by Wang et al. [27].

### 2.2. Experimental Animals and Protocol

Seventy male SPF Sprague Dawley (SD) rats weighing 180–220 g were used in this study. All rats were randomly divided into groups and kept in a room with 3 rats per cage, a temperature of 24–25°C, a humidity of 60%–65%, and a 12-hour light/dark cycle. The animals were given free access to food and sterile drinking water. The experiment was approved by the Medical Ethics Committee of Central South University (Changsha, China, permit no. 2018sydw0198). The rats were acclimatized for one week before the intervention. We randomly allocated 10 rats to the normal group with a normal diet and 60 rats to the diabetic group with a high-fat diet. After 6 weeks, 60 rats in the diabetic group were fasted for 8 hours and then injected with 35 mg/kg STZ intraperitoneally. Rats in the control group were intraperitoneally injected with an equal volume of sodium citrate-citric buffer. Rats with fasting blood glucose (FBG) ≥16.7 mol/L for 3 consecutive days were considered to be a successful T2DM rat model [19, 27].

Finally, 57 T2DM rats in the diabetic group were randomly allocated into 6 groups: the model group (*n* = 10), GGJTW group (*n* = 10), fecal transplant group (T group, *n* = 10), metformin group (Met group, *n* = 7), antibiotics group (Abs group, *n* = 10), and GGJTW + antibiotics group (GA group, *n* = 10). Ten rats in the control group served as the normal group. According to our previous study, we found that treating diabetic rats with 8.1 g/kg/day GGJTW can relieve the symptoms of diabetes to a great extent [27]. Therefore, rats in the GGJTW group and GA group were treated with 8.1 g/kg/day GGJTW by gavage. The Met group was treated with 200 mg/kg/day metformin with the same method [27]. The T group was treated with 5 g/kg/day feces (collected from the GGJTW group) by gavage. The normal model and Abs groups were given an equal volume of saline. Meanwhile, pseudogerm-free rats, including the Abs group and GA group, were treated with vancomycin 25 *μ*g/mL, metronidazole 100 *μ*g/mL, and 50 *μ*g/mL neomycin in sterile water per day [19, 28]. After intervening for six weeks, the rats were fasted for 12 hours and anesthetized with 2% isoflurane inhalation, and blood samples were collected through abdominal aortic puncture. The rats were dissected, and the colon contents and tissues were collected under aseptic conditions.

### 2.3. Biochemical Assays

A glucometer (Roche Diagnostics Co., Ltd., Germany) was used to measure rat tail vein blood FBG once a week. After 6 weeks of drug treatment, all rats were fasted for 8 hours and gavaged with 2.0 g/kg D-glucose (25% solution). Then, blood glucose was measured at 0 min, 5 min, 10 min, 30 min, 60 min, and 120 min. We multiplied each blood glucose value by time to calculate the area under the curve (AUC). An automatic biochemical analyzer was used to immediately detect serum TG, TC, LDL-C, and HDL-C.

### 2.4. Enzyme-Linked Immunosorbent Assay (ELISA)

GLP-1 and fasting blood insulin (FINS) were quantified by using ELISA kits (GPL-1, Elabscience, Wuhan, China; FINS: Merck Millipore Co., Ltd, Darmstadt, Germany), and the index of homeostasis assessment of insulin resistance (HOMA-IR) was calculated by the following formula: HOMA-IR = FBG (mmol/l) × FINS (mU/l)/22.5.

### 2.5. DNA Extraction and 16S rRNA Gene Amplification and Sequencing

We randomly selected 6 samples of colon contents from each group for intestinal flora analysis. A TIANamp Stool DNA Kit was used to extract microbial DNA from the colon contents (DP328, TIAGEN Biotech Co., Ltd., China), following the manufacturer's instructions. The final concentration and purity of DNA were determined by using a NanoDrop (NC2000, Thermo Scientific) and 1% agarose gel electrophoresis. The V3-V4 regions of the bacterial 16S rRNA gene were amplified with a set of primers: 338F: 5-ACTCCTACGGGAGGCAGCAG-3, 806R: 5′-GGACTACHVGGGTWTCTAAT-3′, and PCR amplification was carried out in a PCR system (ABI GeneAmp® 9700, USA). The AxyPrep DNA Gel Extraction Kit (AP-GX-500, Axygen Biosciences Co., Ltd., USA) was used to purify the PCR products, and the QuantiFluor™-ST (Promega Co., USA) was used for quantification. Later, we used the TruSeqTM DNA Sample Preparation Kit (FC-121-4001, Illumina, USA) to construct the sequencing library. The microbiomes were analyzed at the Shanghai Majorbio Bio-Pharm Technology Co., Ltd., on an Illumina MiSeq platform.

### 2.6. Bioinformatics Analysis

Trimmomatic software was used for quality control of the raw sequences, and FLASH software was used to assemble the paired-end reads. With a 97 % similar threshold, the operational taxonomic units (OTUs) were selected. All of the OTUs were put into the Silva 16S rRNA database for taxonomy at the domain, kingdom, phylum, class, order, family, genus, and species levels. Principal coordinates analysis (PCoA) was used to observe the similarities and differences between different groups at the OTU level. The ace index and the Shannon index, which are commonly used to estimate the total number of species in ecology and reflect the diversity of gut community, were calculated by the mothur software. The rank-abundance curve is another method to reflect the diversity of the gut microbiota and was drawn with *R* software. A rarefaction curve, which was drawn with the Shannon index at the OTU level, was also constructed by *R* software. Later, we observed the changes in gut bacteria at the phylum and the genus levels. The composition of the gut bacteria, the ratio of *Firmicutes*/*Bacteroidetes,* and the relative abundance of *Spirochaetea and Proteobacteria* were demonstrated. The top 50 most abundant genera were selected; then, the z-score method was used to normalize their abundance. Subsequently, Student's *t*-test was used to compare the model and GGJTW groups; if the *P* value was less than 0.1, this genus was selected for further analysis. Finally, 19 genera were selected for building Spearman's correlation coefficient with biomarkers by using SPSS 24.0.

### 2.7. LC-MS Analysis of PBAs in Colon Contents

First, we added 100 mg colon contents to 500 *μ*L of acetonitrile. Second, the mixed sample was vortexed for 60 seconds, placed statically for 20 minutes (−20°C), and centrifuged for 20 minutes (4°C, 14000 g). Finally, the supernatant was collected for later use. The abovementioned steps were repeated 3 times. We used the Waters ACQUITY UPLC I-Class system for UHPLC analysis and 5500 QTRAPs (AB, SCIEX) for MS analysis. Six PBAs were weighted accurately. Eight different concentrations of each PBAs were prepared with acetonitrile. Then, the standard solutions were measured with LC/MS.  Table 1 provides the standard curves of PBAs. According to the peak areas and standard curves, the concentrations of PBAs were quantified.

### 2.8. Quantitative Real-Time PCR (qRT-PCR)

The colon tissues of the rats were stored at −80°C, and total RNA was isolated from the frozen tissues with Trizol reagent. We synthesized the cDNA from 1 *μ*g total RNA using a Reverse Transcription Kit (K1622, Thermo Scientific, USA) [5, 19]. An SYBR green PCR master mix was used for quantitative reverse transcriptase PCR analysis (Monad Biotech Co., Ltd., China). The analysis was performed by using a Bio-Rad CFX Connect PCR system (Bio-Rad, USA). Primers of FXR and TGR5 and GAPDH are listed in Table 2. The 2(^−ΔΔCt^) method was performed for relative gene expression analysis.

### 2.9. Statistical Analysis

All data are expressed as the mean ± SD, and SPSS 23.0 and GraphPad Prism 7.0 software were used for statistical analysis and graphical presentation. One-way ANOVA was performed to analyze the alterations of FBG, AUC, HOMA-IR, serum lipids, and bile acids, mRNA expression of FXR and TGR5, and the concentration of GLP-1. LSD was used for the post hoc test. Student's *t*-test was performed to determine the relative abundance of intestinal flora between the model group and every other group at the genus level. *P*<0.05 was considered statistically significant.

## 3. Results

### 3.1. Effects of GGJTW on FBG, OGTT, and HOMA-IR

**Figure 1:**
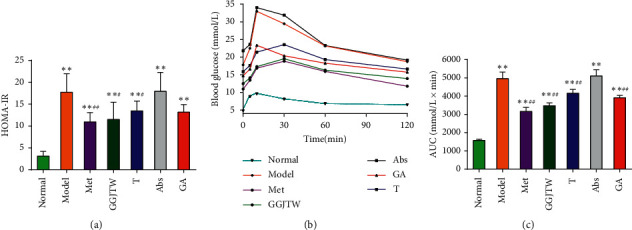
(a) HOMA-IR; (b) OGTT; (c) AUC. Met group, *N* = 7, and the other groups, *N* = 10. ^*∗*^*P* < 0.05, ^*∗∗*^*P* < 0.01 to the normal group; ^#^*P* < 0.05, ^##^*P* < 0.01 to the model group. One-way ANOVA was used for statistical analysis.

Our previous study demonstrated that GGJTW significantly reduces FBG, and this study agreed with the abovementioned results [27]. The dynamic monitoring of FBG is presented in Table 3. We observed that GGJTW significantly reduced FBG from the second week and reached a peak in the sixth week. The FBG of fecal transplant rats (T group) also decreased slowly and was reduced by 19.4% at week 6 (*P* < 0.01), indicating that the gut microbiota changed by GGJTW has a hypoglycemic effect. Compared with the model group, the FBG in the GA group was reduced but not as significant as that of the GGJTW group. These results indicated that the hypoglycemic effect is related to the gut microbiota but does not only rely on this factor. Compared with the normal group, the HOMA-IR index in the model group showed a significant increase, while the GGJTW treatment group showed a significant decrease (Figure 1(a)). The HOMA-IR of the fecal transplant group also decreased compared to that of the model group but was lower than that of the GGJTW group (Figure 1(a)). GGJTW also reduced HOMA-IR when the gut microbiota of diabetic rats was disturbed by antibiotics (Figure 1(a)). The results of the OGTT confirmed that GGJTW could improve glucose intolerance, and this result was also observed in the fecal transplant group, with the AUCs decreasing by 29.9% and 16.1%, respectively (Figure 1(b) and 1(c)). Blood glucose levels also decreased in the GA group, and the AUC was reduced by 21.1% compared to the model group (Figure 1(b) and 1(c)).

Values are means ± SD, *N* = 7 in the Met group, and *N* = 10 in the other groups. Statistical analysis was performed by one-way ANOVA. ^*∗*^*P* < 0.05, ^*∗∗*^*P* < 0.01 vs. normal; ^#^*P* < 0.05, ^##^*P* < 0.01 vs. model; and ^ΔΔ^*P* < 0.01 vs. Abs.

### 3.2. Effects of GGJTW on Serum Lipids

**Figure 2:**
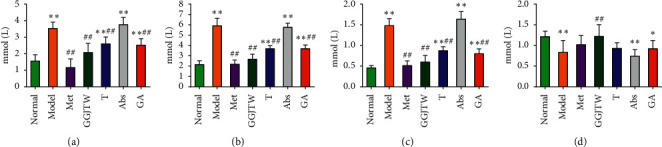
GGJTW improved dyslipidemia in the type 2 diabetic rats. Met group *N* = 7, and the other groups, *N* = 10. ^*∗*^*P* < 0.05, ^*∗∗*^*P* < 0.01 to the normal group; ^#^*P* < 0.05, ^##^*P* < 0.01 to the model group. One-way ANOVA was used for statistical analysis. (a) TG. (b) TC. (c) LDL-C. (d) HDL-C.

A significant difference in blood lipid levels was detected between the model group and the GGJTW group, indicating that GGJTW could improve dyslipidemia in the type 2 diabetic rats. Compared to the model group, TG, TC, and LDL-C in the GGJTW group were decreased by 41.2%, 54.9%, and 59.5%, respectively, while HDL-C was increased by 46.7%. The serum lipids in the fecal transplant rats were also improved, and TG, TC, and LDL-C were decreased by 26.2%, 37.6%, and 41.1%, respectively. HDL-C was increased by 11.5%, but there was no significant difference compared to the model group. A similar trend was observed in the GA group, with TG, TC, and LDL-C reduced by 28.4%, 37.5%, and 46.1% and HDL-C slightly increased by 10.4%. The serum lipids in the different groups are shown in Figure 2.

### 3.3. Influence of GGJTW on the Gut Microbiota Diversity and Richness

**Figure 3:**
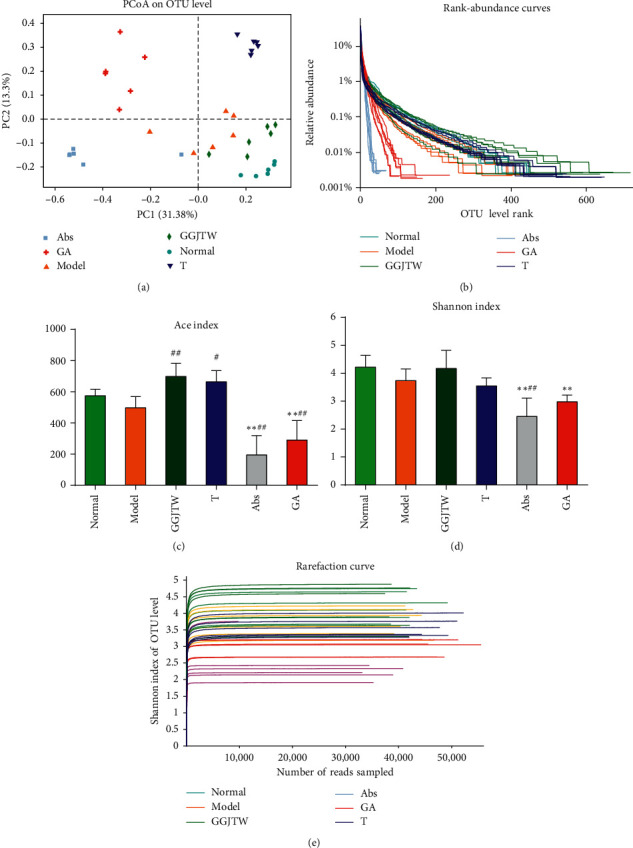
(a) PCoA in different groups; (b) rank-abundance curve; (c) ace index; (d) Shannon index; and (e) rarefaction curve; *N* = 6. ^*∗*^*P* < 0.05, ^*∗∗*^*P* < 0.01 to the normal group; ^#^*P* < 0.05, ^##^*P* < 0.01 to the model group. One-way ANOVA was used for statistical analysis.

In total, 1046 OTUs were obtained from 36 colon content samples. PCoA was performed for the whole dataset to view the clustering trend of the samples with multidimensional data. As shown in Figure 3(a), a clear separation between the model group and the normal group was observed, suggesting that the gut microbiota of T2DM rats and normal rats were different from each other. Furthermore, we observed that all of the groups contributed to maintaining a unique cluster, suggesting that every group in our study had a unique composition of gut microbes at the OTU level (Figure 3(a)). A rank-abundance curve was used to analyze the abundance and uniformity of intestinal flora at the OTU level. As shown in Figure 3(b), rats in the normal, GGJTW, and T groups had a relatively higher diversity, and rats in the Abs and GA groups had the lowest diversity. To further observe the influence of GGJTW on the diversity and richness of the gut microbiota, the ace index and Shannon index were calculated. Compared to the model group, GGJTW and fecal transplant significantly improved the ace index (Figures 3(c)–3(d)). Antibiotics eliminated most of the gut microbiota; therefore, rats in the Abs group and GA group had the lowest ace index and Shannon index (Figures 3(c)–3(d)). The rarefaction curve verified the abovementioned results and demonstrated that the sample size in our study was reasonable (Figure 3(e)).

### 3.4. GGJTW Altered the Gut Microbiota Composition at the Phylum Level

**Figure 4:**
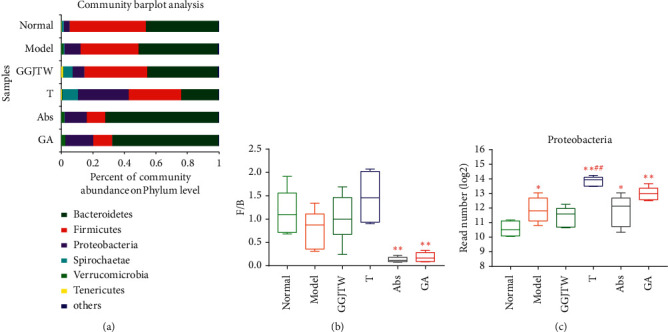
(a) Bacterial composition at the phylum level; (b) *Firmicutes/Bacteroidetes* ratio; and (c) the read number (log2) of *Proteobacteria*. *N* = 6. ^*∗*^*P* < 0.05, ^*∗∗*^*P* < 0.01 vs. normal; ^#^*P* < 0.05, ^##^*P* < 0.01 vs. model. One-way ANOVA was used for statistical analysis.

To study the specific changes in gut flora, we tested the relative abundance of the main taxonomic groups at the phylum level in a different group. As shown in Figure 4(a), every group was abundant with *Bacteroidetes*, *Firmicutes,* and *Proteobacteria*, but the composition ratio was different. The relative abundance of B*acteroidetes* was the highest in the Abs and GA groups, with 71.8% and 67.5%, respectively, 44.7% in the GGJTW group, and only 23.6% in the fecal transplant group (Figure 4(a)). In contrast, *Firmicutes* was abundant in the normal and GGJTW groups, with 48.6% and 40.0%, respectively, and only 11.7% and 12.0% in the Abs and GA groups, respectively. The ratio of *Firmicutes* to *Bacteroidetes* (F/B) was calculated. The GGJTW and fecal transplant groups had an increased F/B ratio, but there was no significant difference compared to the model group, and the Abs and GA groups had an obviously reduced F/B ratio (Figure 4(b)). The relative abundance of *Proteobacteria* was the highest in the fecal transplant group, at 31.8%, and it was only 3.8% in the normal group, 7.5% in the GGJTW group, and 10.2% in the model group (Figures 4(a)–4(c)). The relative abundance of *Proteobacteria* in the Abs and GA groups was 13.9% and 17.6% (Figures 4(a)–4(c)). These results indicate that GGJTW and antibiotics could significantly change the gut microbiota at the phylum level.

### 3.5. GGJTW Altered the Gut Microbiota Composition at the Genus Level

**Figure 5:**
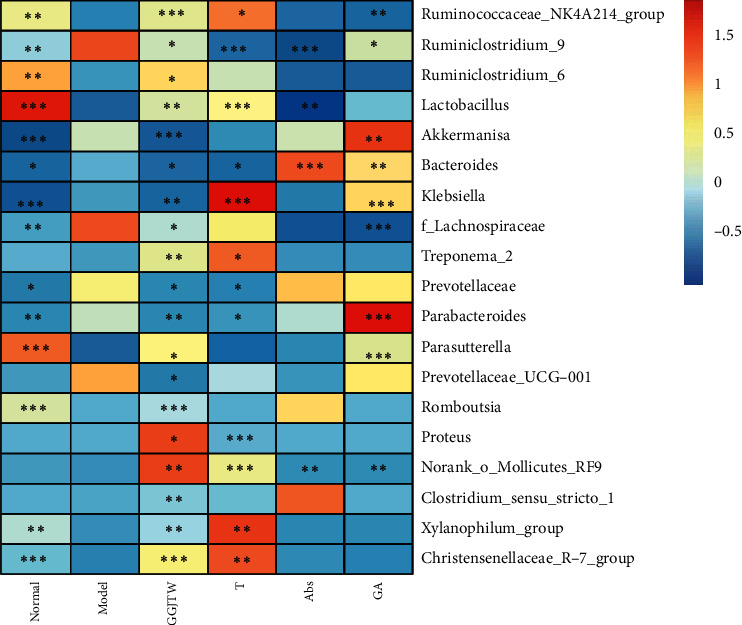
Heatmap of the differential bacteria in the top 50 most abundant genera. The data were normalized with the z-score method. Student's *t*-test was conducted between the model group and every other group. ^*∗*^*P* < 0.1, ^*∗∗*^*P* < 0.05, and ^*∗∗∗*^*P* < 0.01.

By analyzing the abundance of the intestinal flora, we found that the composition of the flora changed at the genus level. Then, 50 genera with the richest abundance were selected. Based on our selected threshold (*P* < 0.1, GGJTW group vs. model group), 19 genera were identified as the final observed genera (Figure 5). Compared to the model group, the relative abundance of 11 genera was increased in the GGJTW group. Except for *Klebsiella* and *Romboutsia*, the genera in the fecal transplant group had the same changes as those in the GGJTW group. Compared to the normal group, most of the genera were greatly decreased after intervention with antibiotics. Many studies have found that *Lactobacillus* are beneficial for T2DM treatment [29–31] and the production of bile acids [25, 32]. In our study, *Lactobacillus* was increased not only in the GGJTW and fecal transplant groups but also in the GA group. *Akkermansia* and *Parabacteroides* are beneficial microbes [33, 34] and were not increased in the GGJTW or fecal transplant groups but were significantly increased in the GA group (Figure 5).

### 3.6. The Relationship between Selected Genera and Biomarkers

**Figure 6:**
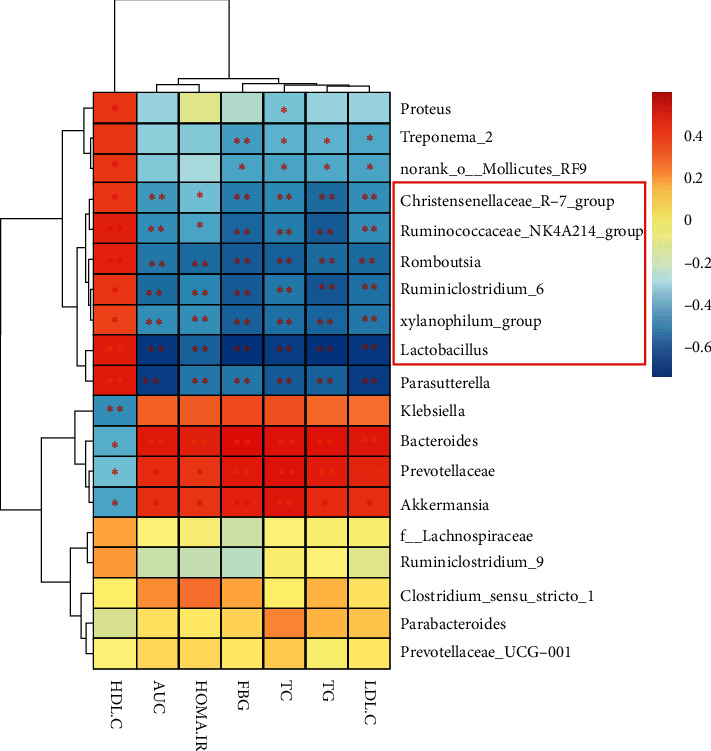
Spearman's correlations of selected differential genera with the biomarkers. Genera in the red frame belong to *Firmicutes*. ^*∗*^*P* < 0.05; ^*∗∗*^*P* < 0.01.

To explore the associations of the gut microbiota with biomarkers, Spearman's correlation coefficient between 19 selected genera and biomarkers was calculated, and the results are shown in Figure 6 and Table 4. Ten genera were found to be negatively related to AUC, HOMA-IR, FBG, TC, TG, and LDL-C and positively related to HDL-C, and these bacteria may be promising for treating T2DM. However, contrary results were observed for *Klebsiell*a, *Bacteroides*, *Prevotellaceae,* and *Akkermansia*, and these bacteria may be ineffective for treating T2DM. Five genera were slightly positively correlated with all of the biomarkers. Moreover, we found that 6 genera, including the *Christensenellaceae_R-7_group*, *Ruminococcaceae_NK4A214_group*, *Romboutsia*, *Ruminiclostridium_6*, *xylanophilus,* and *Lactobacillus*, belonged to *Firmicutes*, which are beneficial for treating T2DM (Table 4). The PBA-related bacterium *Lactobacillus* [32] was strongly negatively correlated with FBG (*r* = −0.690, *P* = 3.252*E*−06), AUC (*r* = −0.669, *P* = 8.06*E*−06), HOMA-IR (*r* = −0.561, *P* = 3.725*E*−04), TG (*r* = −0.695, *P* = 2.603*E*−06), TC = (*r* = −0.650, *P* = 1.755E−05), and LDL (*r* = −0.668, *P* = 8.680*E*−06) and positively correlated with HDL-C (*r* = 0.463, *P* = 4.429*E*−03).

### 3.7. GGJTW Protects against T2DM via the PBA-FXR/TGR5-GLP-1 Pathway

**Figure 7:**
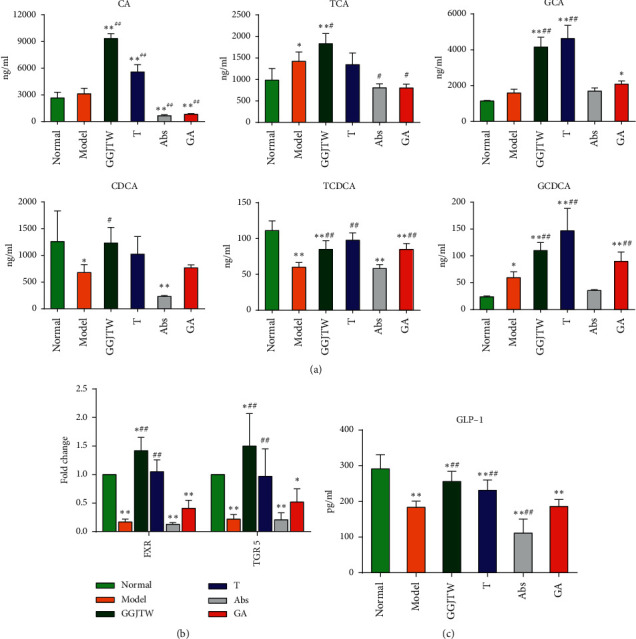
(a) The concentration of PBAs in colon contents; (b) the relative expression of FXR and TGR5 in the colon tissue; and (c) the concentrations of GLP-1 in serum. *N* = 6, ^*∗*^*P* < 0.05, ^*∗∗*^*P* < 0.01 vs. normal; ^#^*P* < 0.05, ^##^*P* < 0.01 vs. model. One-way ANOVA was used for statistical analysis.

Our previous untargeted metabolomics study found that PBAs in serum were significantly increased after GGJTW treatment. In this study, we detected PBA in the colon contents by LC/MS, and six PBAs, CA, TCA, GCA, CDCA, TCDCA, and GCDCA were upregulated in the GGJTW group (Figure 7(a)). The concentration of PBAs in the T group was increased. Compared to the Abs group, PBAs in the GA group were also improved. FXR and TGR5 are receptors of PBAs. Compared to the model group, the expression levels of FXR and TGR5 mRNA were significantly upregulated in the GGJTW, T, and GA groups (Figure 7(b)). GLP-1 is beneficial for the treatment of T2DM, and the secretion of GLP-1 is associated with the activities of FXR and TGR5. Hence, we detected the concentration of GLP-1 in serum. Consistent with these findings, the concentration of GLP-1 was increased after GGJTW treatment (Figure 7(c)).

## 4. Discussion

In the present study, GGJTW treatment of STZ-induced diabetic rats significantly decreased the FBG from the second week. Moreover, improvements in insulin sensitivity, glucose homeostasis, and lipid metabolism in T2DM rats were observed (Figures 1 and 2). The therapeutic effects of GGJTW on diabetes were in good agreement with our previous studies [27]. To study the effects of GGJTW on the gut microbiota for the treatment of T2DM, antibiotics were administered to the diabetic rats, and we found that FBG and other diabetes-related parameters were improved in the GA group, but were weaker than those in the GGJTW group. Moreover, feces from the GGJTW group rats were transplanted to T2DM rats, and the symptoms of diabetic rats in the fecal transplant group were slightly alleviated. This study strongly verified that GGJTW could lower blood glucose, improve insulin sensitivity, maintain glucose homeostasis, and regulate lipid disorders, and the mechanism of GGJTW in treatingT2DM is closely related to gut microbiota.

A unique cluster of each group was observed in the PCoA picture, suggesting that both GGJTW and antibiotics significantly changed the gut microbiota (Figure 3). Diversity and richness analysis at the OTU level revealed that the GGJTW and fecal transplant groups could increase the ace index and Shannon index, and these two indexes were significantly decreased in the antibiotics group. The rank-abundance and rarefaction curves verified abovementioned results.

The results of this study showed that, at the phylum level, *Bacteroides*, *Firmicutes,* and *Proteobacteria* were the dominant phyla in each group (Figure 4). Many studies have demonstrated that compared with nondiabetic patients, the abundance of *Firmicutes* and the F/B ration in diabetic patients is reduced [35–37]. However, *Proteobacteria* and *Bacteroidetes* are more abundant in T2DM subjects [38, 39]. Some studies found that increasing the F/B ration is helpful for the treatment of T2DM [12, 40]. In our study, we revealed that the relative abundance of *Firmicutes* was decreased in diabetic rats and increased in GGJW-treated and GGJTW-based fecal transplant rats, which agrees with the abovementioned studies. In addition, the ratio of *Bacteroidetes* increased in diabetic rats, especially in antibiotic-treated rats, and this phenomenon might result in the *Bacteroides* having the most antibiotic-resistant bacteria and the highest resistance rates of all anaerobic bacteria [41]. Otherwise, GGJTW reduced the relative abundance of *Bacteroidetes*, which was increased by antibiotic intervention. Correspondingly, the F/B ration was slightly increased in the GGJTW and T groups and decreased in the Abs and GA groups. According to previous studies, *Proteobacteria* is one of the common pathogens of human diseases. Our study found that GGJTW can reduce the relative abundance of *Proteobacteria*. Because *Proteobacteria* have a stronger ability to colonize [42], the results were inconsistent between the GGJTW group and the GGJTW-based fecal transplant group rats, and the fecal transplant group had the highest ratio of *Proteobacteria* compared with the lowest in the GGJTW-treated rats.

Based on our primary research, PBAs including CA, CDCA, TCA, GCA, and TCDCA were upregulated by GGJTWin serum [27].Our study reconfirmed this result and ultimately revealed that the regulation of PBAs by GGJTW is correlated with gut microbiota (Figure 7). A study found that an increase in *Firmicutes* promotes the metabolism of bile acids [43]. Consistently, the composition of *Firmicutes* was significantly increased in the GGJTW-treated and GGJTW-based fecal transplant groups, accompanied by a higher F/B ratio (Figure 4). Moreover, an analysis of the composition at the genus level revealed significant upregulation of the probiotic *Lactobacillus* in GGJTW-treated rats (Figure 5). Similarly, Spearman's correlation analysis found that 6 beneficial T2DM genera (including the *Christensenellaceae_R-7_group*, *Ruminococcaceae_NK4A214_group*, *Romboutsia*, *Ruminiclostridium_6*, *xylanophilus,* and *Lactobacillus*) belonging to *Firmicutes* (Figure 6), were highly correlated with the diabetic biochemistry parameters.

For a long time, it was believed that *Lactobacillus* is beneficial to human health, and it was isolated from the entire gastrointestinal tract, skin, and vagina [31].More than 200 *Lactobacillus* species were isolated, and over 50 species were repeatedly detected in the stools of healthy volunteers [31, 44]. A mass of evidence revealed that *Lactobacillus* species have numerous therapeutic abilities, including anticancer, cholesterol lowering, antioxidant, antibacterial, antiviral, antidiarrhea, and treating T2DM [45]. Furthermore, *Lactobacillus* species were considered to be bile-acid-producing bacteria [32]. In our study, *Lactobacillus* was increased not only in the GGJTW and fecal transplant groups but also in the GA group, indicating that the treatment of T2DM might be related to the upregulation of *Lactobacillus*.

PBAs are synthesized in the liver and are transformed into secondary bile acids through the intestinal flora [32]. FXR and TGR5 are the main bile acid receptors [32, 46, 47]. FXR, a nuclear receptor mainly expressed in gut and liver tissues, plays pivotal roles in maintaining the homeostasis of BAs, lipids, glucose, and energy [48]. A study found that *Fxr-/-* mice exhibit increased serum glucose and impaired glucose and insulin tolerance [49]. The activation of FXR could inhibit glycolysis and elevate the secretion of GLP-1, suggesting that FXR might be a promising therapeutic target for T2DM [50]. TGR5 is a *G*-protein-coupled bile acid receptor and a key regulator of glucose homeostasis [51]. Several bile acids, including lithocholic acid (LCA), DCA, CDCA, and CA, have the ability to activate TGR5 [51]. Tgr5−/− mice have impaired glucose tolerance and insulin resistance [52]. Due to TGR5-mediated effects on GLP-1 secretion, TGR5 is recognized as a potential target for the treatment of metabolic disorders [51].

In our study, not only PBAs but also the mRNA expression levels of FXR and TGR5were upregulated. The activation of PBA-FXR/TGR5 could induce the release of GLP-1 from intestinal *L* cells [45–47], and upregulated expression of GLP-1 promotes insulin secretion, increases insulin sensitivity, and reduces gluconeogenesis [53]. Consistent with the abovementioned results, GLP-1 was significantly improved in the GGJTW- and GGJTW-based fecal transplant groups, and after intervention with antibiotics, the improvement in GLP-1 was depressed.

In summary, the results of our study revealed that GGJTW could ameliorate hyperglycemia and hyperlipidemia in T2DM rats and provide a reliable support for the clinical application of GGJTW. Furthermore, we found that its therapeutic effects are related to the gut microbiota. Gut microbiota composition analysis revealed that the diabetic beneficial phylum *Firmicutes* and bile acid-related genus *Lactobacillus* were markedly increased after treatment with GGJTW. Consistent with our previous study, the increase in PBAs was reconfirmed. In the GGJTW treatment group, PBAs promoted the activation of FXR/TGR5 and promoted the secretion of GLP-1, which is highly beneficial for the treatment of T2DM. The findings of this study suggest that GGJTW exerts hypoglycemic effects predominantly via the gut-microbiota-mediated PBA pathway.

## Figures and Tables

**Table 1:** Standard curves of primary bile acids.

Component name	Mass information	Retention time	Linear	*R* ^2^
CA	407.4/407.4	8.041582512	*y* = 0.00702*x* − 0.03110	0.99942
CDCA	391.4/391.4	11.17777284	*y* = 0.01398*x* + 0.12827	0.99961
TCA	514.4/79.9	4.404032604	*y* = 0.00573*x* − 0.03420	0.99898
GCA	464.4/74.0	5.470159547	*y* = 0.01117*x* − 0.03901	0.99938
TCDCA	498.4/80.0	6.686601991	*y* = 0.00475*x* + 0.00137	0.99995
GCDCA	448.4/74.0	7.996163695	*y* = 0.02906*x* + 0.00967	0.99999

**Table 2:** Specific primers for the qRT-PCR analysis.

Gene	Forward	Reverse
FXR	AGGATAGAGAGGCAGTGGAGAAGC	AGCGTGGTGATGGTTGAATGTCC
TGR5	GCCTCATCGTCATCGCCAACC	TTAGAAAGAAGCAGCCAGCAGGTG
GAPDH	GCCATCAACGACCCCTTCAT	TCCCGTTGATGACCAGCTTC

**Table 3:** Dynamic FBG in different groups (mmol/L).

	0 w	1 w	2 w	3 w	4 w	5 w	6 w
Normal	4.63 ± 0.34	4.42 ± 0.39	4.37 ± 0.45	4.65 ± 0.50	4.52 ± 0.47	4.56 ± 0.27	5.07 ± 0.49
Model	19.33 ± 2.50^*∗∗*^	18.51 ± 3.12^*∗∗*^	19.55 ± 2.35^*∗∗*^	19.95 ± 2.70^*∗∗*^	18.21 ± 3.02^*∗∗*^	17.34 ± 2.48^*∗∗*^	18.17 ± 1.80^*∗∗*^
Met	19.24 ± 1.52^*∗∗*^	15.84 ± 2.52^*∗∗*^	15.01 ± 2.48^*∗∗*^^##^	11.27 ± 2.10^*∗∗*^^##^	11.89 ± 3.01^*∗∗*^^##^	10.40 ± 3.60^*∗∗*^^##^	10.73 ± 2.11^*∗∗*^^##^
GGJTW	19.24 ± 1.64^*∗∗*^	17.57 ± 1.71^*∗∗*^	16.04 ± 1.95^*∗∗*^^##^	14.66 ± 2.14^*∗∗*^^##^	13.27 ± 1.19^*∗∗*^^##^	12.47 ± 1.56^*∗∗*^^##^	12.39 ± 1.54^*∗∗*^^##^
T	19.24 ± 1.44^*∗∗*^	18.89 ± 1.91^*∗∗*^	17.99 ± 1.58^*∗∗*^	16.94 ± 1.23^*∗∗*^^#^	16.05 ± 1.33^*∗∗*^	15.42 ± 1.51^*∗∗*^^#^	14.64 ± 1.81^*∗∗*^^##^
Abs	19.35 ± 1.36^*∗∗*^	20.01 ± 1.63^*∗∗*^	20.71 ± 2.15^*∗∗*^	21.12 ± 2.54^*∗∗*^	20.48 ± 2.86^*∗∗*^	21.24 ± 2.17^*∗∗*^^#^	21.03 ± 2.88^*∗∗*^^#^
GA	19.33 ± 1.71^*∗∗*^	18.64 ± 1.35^*∗∗*^	17.67 ± 1.44^*∗∗*^^ΔΔ^	16.48 ± 1.21^*∗∗*^^##ΔΔ^	15.91 ± 1.51^*∗∗*ΔΔ^	14.93 ± 1.85^*∗∗*#ΔΔ^	14.68 ± 2.35^*∗∗*##ΔΔ^

**Table 4:** Spearman's correlations.

Genera	HDL-C	AUC	HOMA-IR	FBG	TC	TG	LDL-C
*Proteus*	0.372^*∗*^	−0.287	−0.107	−0.238	−0.334^*∗*^	−0.289	−0.288
*Treponema*	0.296	−0.304	−0.317	−0.424^*∗∗*^	−0.372^*∗*^	−0.371^*∗*^	−0.389^*∗*^
*norank_o_Mollicutes_RF9*	0.356^*∗*^	−0.323	−0.279	−0.407^*∗*^	−0.412^*∗*^	−0.391^*∗*^	−0.400^*∗*^
*Christensenellaceae_R−7_group*	0.361^*∗*^	−0.433^*∗∗*^	−0.344^*∗*^	−0.487^*∗∗*^	−0.473^*∗∗*^	−0.534^*∗∗*^	−0.457^*∗∗*^
*Ruminococcaceae_NK4A214_group*	0.448^*∗∗*^	−0.449^*∗∗*^	−0.409^*∗*^	−0.535^*∗∗*^	−0.492^*∗∗*^	−0.568^*∗∗*^	−0.462^*∗∗*^
*Romboutsia*	0.443^*∗∗*^	−0.504^*∗∗*^	−0.533^*∗∗*^	−0.574^*∗∗*^	−0.555^*∗∗*^	−0.525^*∗∗*^	−0.528^*∗∗*^
*Ruminiclostridium_6*	0.356^*∗*^	−0.532^*∗∗*^	−0.476^*∗∗*^	−0.574^*∗∗*^	−0.507^*∗∗*^	−0.575^*∗∗*^	−0.524^*∗∗*^
*xylanophilus*	0.331^*∗*^	−0.458^*∗∗*^	−0.455^*∗∗*^	−0.557^*∗∗*^	−0.520^*∗∗*^	−0.542^*∗∗*^	−0.506^*∗∗*^
*Lactobacillus*	0.463^*∗∗*^	−0.669^*∗∗*^	−0.561^*∗∗*^	−0.690^*∗∗*^	−0.650^*∗∗*^	−0.695^*∗∗*^	−0.668^*∗∗*^
*Parasutterella*	0.484^*∗∗*^	−0.657^*∗∗*^	−0.512^*∗∗*^	−0.502^*∗∗*^	−0.573^*∗∗*^	−0.551^*∗∗*^	−0.646^*∗∗*^
*Klebsiella*	−0.452^*∗∗*^	0.253	0.267	0.297	0.291	0.244	0.215
*Bacteroides*	−0.376^*∗*^	0.476^*∗∗*^	0.453^*∗∗*^	0.556^*∗∗*^	0.505^*∗∗*^	0.521^*∗∗*^	0.488^*∗∗*^
*Prevotellaceae*	−0.340^*∗*^	0.396^*∗*^	0.360^*∗*^	0.479^*∗∗*^	0.520^*∗∗*^	0.473^*∗∗*^	0.433^*∗∗*^
*Akkermansia*	−0.413^*∗*^	0.393^*∗*^	0.367^*∗*^	0.440^*∗∗*^	0.494^*∗∗*^	0.404^*∗*^	0.394^*∗*^
*Lachnospiraceae*	0.145	−0.078	−0.058	−0.170	−0.078	−0.054	−0.042
*Ruminiclostridium9*	0.160	−0.176	−0.192	−0.219	−0.048	−0.073	−0.123
*Clostridiumsensustricto1*	−0.005	0.176	0.235	0.149	−0.010	0.118	0.007
*Parabacteroides*	−0.134	0.011	−0.002	0.052	0.187	0.124	0.093
*PrevotellaceaeUCG001*	−0.082	0.037	0.036	0.000	0.066	−0.021	0.002

^*∗*^ *P* < 0.05, ^*∗∗*^*P* < 0.01.

## Data Availability

The data used to support the findings of this study are included within the article.
